# Supplementary material for: Computational insights on the hydride and proton transfer mechanisms of L-proline dehydrogenase
Source: PLoS One. 2023 Nov 15;18(11):e0290901. doi: 10.1371/journal.pone.0290901 (PMC10651016; doi:10.1371/journal.pone.0290901)
Supplement: S1 File — (DOCX) [file pone.0290901.s001.docx]

**Computational Insights on the Hydride and Proton Transfer Mechanisms of L‑Proline Dehydrogenase**

Author(s): Ibrahim Yildiz^†*^

^†^Khalifa University, Chemistry Department and Applied Material Chemistry Center (AMCC), PO Box 127788, Abu Dhabi, UAE Tel: +971 (0)2 401 8208

*E-mail: ibrahim.yildiz@ku.ac.ae

# Supporting Tables

Table S1. Absolute Energies of Reactant Complex, Product Complex, and Transition State for the Hydride Transfer Process with ωB97XD functional with 6-31G basis set (ZPE: Zero-point energy correction, H: Enthalpy, G: Gibbs free energy)

| Species | E+ZPE (au) | H (au) | G (au) |
| --- | --- | --- | --- |
| RC | -1851.9990 | -1851.9629 | -1851.9938 |
| TS | -1851.2997 | -1851.2618 | -1851.2935 |
| PC | -1852.7868 | -1852.7546 | -1852.7835 |

Table S2. Absolute Energies of Reactant Complex, Product Complex, and Transition State for the Hydride Transfer Process with M06-2X functional with 6-31G basis set (ZPE: Zero-point energy correction, H: Enthalpy, G: Gibbs free energy)

| Species | E+ZPE (au) | H (au) | G (au) |
| --- | --- | --- | --- |
| RC | -1851.7732 | -1851.0733 | -1852.5624 |
| TS | -1851.7375 | -1851.0364 | -1852.5293 |
| PC | -1851.7714 | -1851.0713 | -1852.5607 |

Table S3. Absolute Energies of Reactant Complex, Product Complex, and Transition State for the Hydride Transfer Process with CAM-B3LYP functional with 6-31G basis set (ZPE: Zero-point energy correction, H: Enthalpy, G: Gibbs free energy)

| Species | E+ZPE (au) | H (au) | G (au) |
| --- | --- | --- | --- |
| RC | -1851.6308 | -1850.9307 | -1852.4209 |
| TS | -1851.5910 | -1850.8897 | -1852.3829 |
| PC | -1851.6212 | -1850.9207 | -1852.4108 |

Table S4. Absolute Energies of Reactant Complex, Product Complex, and Transition State for the Hydride Transfer Process with M06-2X functional with 6-31G(d,p) basis set (ZPE: Zero-point energy correction, H: Enthalpy, G: Gibbs free energy)

| Species | E+ZPE (au) | H (au) | G (au) |
| --- | --- | --- | --- |
| RC | -1863.1068 | -1852.363469 | -1851.66284 |
| TS | -1863.0587 | -1852.320453 | -1851.619 |
| PC | -1863.0958 | -1852.350947 | -1851.650 |

Table S5. Absolute Energies of Reactant Complex, Product Complex, and Transition State for the Proton Transfer Process with M06-2X functional with 6-31G(d,p) basis set (ZPE: Zero-point energy correction, H: Enthalpy, G: Gibbs free energy)

| Species | E+ZPE (au) | H (au) | G (au) |
| --- | --- | --- | --- |
| RC | -1852.3801 | -1851.6810 | -1853.1628 |
| TS | -1852.3780 | -1851.6793 | -1853.1605 |
| PC | -1852.3762 | -1851.6769 | -1853.1598 |

Table S6. Activation Energies of the proton transfer process with M06-2X functional with 6-31G(d,p) basis set (ZPE: Zero-point energy correction, H: Enthalpy, G: Gibbs free energy, E_af_: Activation energy for the forward reaction, E_ar_: Activation energy for the reverse reaction)

|  | Ea_f_(kcal/mol) | Ea_r_(kcal/mol) |
| --- | --- | --- |
| ΔG | 1.46 | -0.47 |
| ΔE_ZPE_ | 4.32 | 0.39 |
| ΔE | 1.32 | -1.15 |
| ΔH | 1.07 | -1.46 |

Table S7. Activation Energies of the hydride transfer process with M06-2X functional with 6-31G(d,p) basis set (ZPE: Zero-point energy correction, H: Enthalpy, G: Gibbs free energy, E_af_: Activation energy for the forward reaction, E_ar_: Activation energy for the reverse reaction)

|  | Ea_f_(kcal/mol) | Ea_r_(kcal/mol) |
| --- | --- | --- |
| ΔG | 25.28 | 17.67 |
| ΔE_ZPE_ | 30.18 | 23.32 |
| ΔE | 26.99 | 19.14 |
| ΔH | 27.82 | 19.76 |

Table S8. Activation Energies of the hydride transfer process with M06-2X functional with 6-31G basis set (ZPE: Zero-point energy correction, H: Enthalpy, G: Gibbs free energy, E_af_: Activation energy for the forward reaction, E_ar_: Activation energy for the reverse reaction)

|  | Ea_f_(kcal/mol) | Ea_r_(kcal/mol) |
| --- | --- | --- |
| ΔG | 20.79 | 19.70 |
| ΔE_ZPE_ | 25.49 | 25.46 |
| ΔE | 22.43 | 21.30 |
| ΔH | 23.19 | 21.92 |

Table S9. Activation Energies of the hydride transfer process with ωB97XD functional with 6-31G basis set (ZPE: Zero-point energy correction, H: Enthalpy, G: Gibbs free energy, E_af_: Activation energy for the forward reaction, E_ar_: Activation energy for the reverse reaction)

|  | Ea_f_(kcal/mol) | Ea_r_(kcal/mol) |
| --- | --- | --- |
| ΔG | 20.23 | 18.18 |
| ΔE_ZPE_ | 24.97 | 23.10 |
| ΔE | 22.65 | 19.40 |
| ΔH | 23.79 | 19.93 |

Table S10. Activation Energies of the hydride transfer process with CAM-B3LYP functional with 6-31G basis set (ZPE: Zero-point energy correction, H: Enthalpy, G: Gibbs free energy, E_af_: Activation energy for the forward reaction, E_ar_: Activation energy for the reverse reaction)

|  | Ea_f_(kcal/mol) | Ea_r_(kcal/mol) |
| --- | --- | --- |
| ΔG | 23.86 | 17.49 |
| ΔE_ZPE_ | 27.79 | 22.64 |
| ΔE | 25.01 | 18.96 |
| ΔH | 25.72 | 19.47 |

# Supporting Figure

| 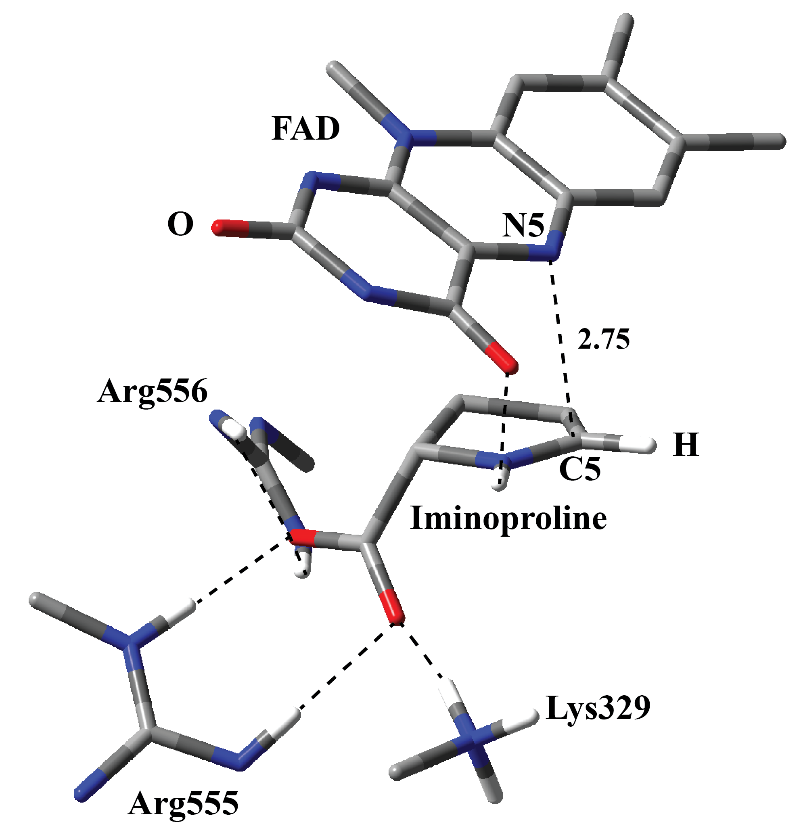 |
| --- |
| **Figure S1**. The structure of optimized PC2 when FADH^-^ is oxidized back to FAD by the loss H atom at N5 position. FAD, iminoproline, Arg555, Arg556, and Lys329 are in QM region obtained with ONIOM(M06-2X /6-31G(d,p):Amber). QM region is shown with tube models. All the H atoms are excluded in the figure for clarity except the ones shown with ivory color. The H bonding interactions and N5-C5 distance are shown with the dashed lines. The distance is in Å. |
