# Supplementary figures and images for: Computational insights on the hydride and proton transfer mechanisms of L-proline dehydrogenase
Source: PLoS One. 2023 Nov 15;18(11):e0290901. doi: 10.1371/journal.pone.0290901 (PMC10651016; doi:10.1371/journal.pone.0290901)

**Table of Contents (TOC)**

**
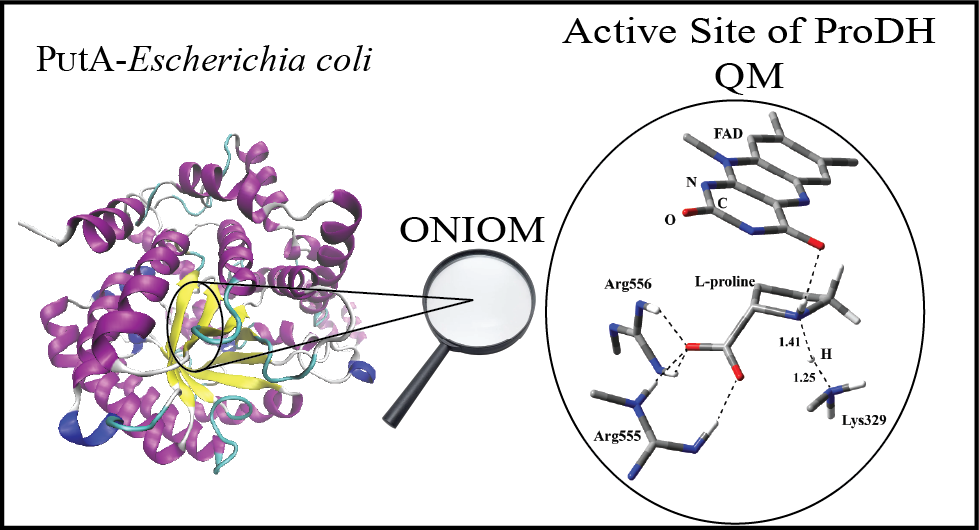
**

Supplement: S1 Graphical abstract — (DOCX) [file pone.0290901.s005.docx]
