# Supplementary material for: Computational insights on the hydride and proton transfer mechanisms of L-proline dehydrogenase
Source: PLoS One. 2023 Nov 15;18(11):e0290901. doi: 10.1371/journal.pone.0290901 (PMC10651016; doi:10.1371/journal.pone.0290901)
Supplement: Attachment — Submitted filename: Response to Reviewers.docx [file pone.0290901.s006.docx]

COMMENTS TO REVIEWERS:

**Dear Reviewers:**

**We would like to express our sincere thanks for your invaluable comments and suggestion for the manuscript. We believe that the manuscript has improved significantly with your suggestions. Below, you will find our responses to each of your comments. We tried to address all suggestions to best of our capacity. Please let us know your further comments.**

**Again thank you so much for your constructive comments.**

**Best wishes,**

Reviewer #1:

Major comments:

(1) The author choose several DFT functionals and basis sets in the calculations. However, the author comes to a conclusion that " the activation barriers in terms of Gibbs free energy with smaller basis set, 6-31G, are better estimates than the values obtained with the larger basis sets. " . This conclution is surprising, because the 6-31G basis set do not gives a proper description to hydrogen, thus larger error would be expected when concerning a reaction with hydrogen element directly involved in TS geometry. More careful calculations should be conducted before the author address this conclusion.

**Author response:**

Our group published several articles using similar protocols for FAD- and NAD-based enzyme reactions particularly hydride and proton transfer reactions using ONIOM method such l-Aspartate Oxidase (ACS Omega 2023, 8, 22, 19963–19968), NOTUM (ACS Omega 2022, 7, 15, 13333–13342), Histone Deacetylase 10 (ACS Omega 2022, 7, 7, 6393-6402), Nicotine Oxidoreductase (ACS Omega, 2021, 6, 22422−22428), L-6-hydroxynicotine (Journal of Molecular Modeling, 2021, 27, 1-13), and Human liver glycerol 3‐phosphate dehydrogenase (Journal of Physical Organic Chemistry 33 (11), e4104). In most of these publications, we have used similar procedures. We have run the calculations with three common DFT functionals with geometry optimizations using 6-31G, 6-31G(d,p=**) basis sets. Also, we performed single point energy calculations using 6311++g(2d,2p) basis set which has both diffuse and polarization functions. We also followed a similar approach in this manuscript. As in the case of previous publications, we have seen that 6-31G functional yielded the lowest activation barrier which is closer to experimentally reported one. We reported this case in these publications. In this revision, we have run calculations using 6-311++G(2d,2p) basis set for three functional and we have seen the same case.

We agree on that 6-31G(d,p) and 6-311++G(2d,2p) basis sets should produce more reliable results than 6-31G basis set. Yet, we don’t have any explanation why 6-31G basis set produce closer activation barriers to experimental one with ONIOM calculations.

(2) The author should give a reasonable explanation about why the bigger basis set singly-point calculations were only performed for M06-2X? Based on so many published benchmark studies, the energies obtained from a small basis set like 6-31G were expected to give a big error, the optimization should be performed at least on 6-31G** level, and Single-Point calculations for other reported functionals are required.(3) For the description of "The activation barriers for hydride transfer process in terms of absolute energy are 3-10 kcal/mol more than in terms of Gibbs free energy indicating that enthalpic and entropic terms lower the activation barrier. ". The affect of the enthalpic and entropic correction can be directly read from the log file of a frequency calculation, the author should provide exact numbers at here.

**Author response:**

As suggested, we have calculated the activation barriers for both hydride and proton transfer steps with 6-311++G(2d,2p) basis set for other two functionals and updated Table 1. Activation energies in terms of absolute energy, zero-point corrected absolute energy, enthalpy, and Gibbs free energy are provided in the supplementary document.

Minor comments:

(1) Too many background information about the PRODH was mentioned in the Abstract section of the manuscript.

**Author response:**

As suggested, we have simplified the abstract.

Reviewer #2: The authors provide a well done computational study of the mechanism of proline oxidation by the flavin-dependent enzyme proline dehydrogenase. The findings will be of significant interest to the field as the results provide unique insights into mechanistic possibilities for ProDH. The manuscript is well written and the results are explained in a succinct manner. Some recommendations are suggested below.

Comments1. Abstract- Line 1: Replace “member of amino acid oxidase” with is “flavin-dependent oxidoreductase”

**Author response:**

As suggested, it was replaced.

2. Abstract- line 14: Replace “supported” with “support”

**Author response:**

As suggested, it was replaced.

3. Page 2, line 3 from the bottom: Replace “L-proline oxidation is followed by the reduction of FAD through ubiquinone.” With “The first half of the reaction involves L-proline oxidation and formation of the reduced FAD cofactor, which is then oxidized by ubiquinone in the second half of the reaction.”

**Author response:**

As suggested, it was replaced.

4. Page 2, line 4 from the bottom: Replace “involving the activity of ProDH” with “from investigating the ProDH activity”

**Author response:**

As suggested, it was replaced.

5. Page 7, line 13 “L-proline binds to the active site”

**Author response:**

As suggested, “the” was added.

6. Figure 4 shows the modeling of proline in active site with the amino group protonated. Did the authors try modeling proline without the amino group protonated? Or can only the protonated form of proline be modeled active site?

**Author response:**

Many experimental studies concluded that amino groups of amines and aminoacids bind to the active site of amine oxidases as their amino groups protonated. Following the binding, an active site base deprotonates the amine group. For ProdH, this base was proposed to be Lys329. For that reason, we modeled the active site as L-proline’s amino group protonated and Lys329 side amino group as neutral. Following the proton transfer step, L-proline’s amino group becomes neutral and Lys329’s amino group becomes positive.

7. Provide labels for the Figures. For example, in Figure 5 label panel A as “TS1” and panel B as “PC1”. Figure 6, panel A “TS2” and panel B “PC2”.

**Author response:**

As suggested, it was done.

8. The distance between the N-atom of Lys and amino group of proline is calculated to decrease upon going from RC1 and TS1. Is there also a change in distance between the carbonyl O atom of the FAD and the proline amino group between RC1 and TS1?

**Author response:**

The distance between the carbonyl O atom of the FAD and the proline amino group in RC1 is 2.92 Å. The same distance is 2.97 Å in TS1. There is a slight increase in the distance.

9. The authors provide an analysis of the different energy barriers for the proton and hydride transfer steps. Can the binding energies of the substrate/product molecules in the RC1, TS1 and PC1 also be estimated from the modeling?

**Author response:**

From RC1, TS1, and PC1 the substrate binding energy cannot be calculated. These states belong to enzyme with bound substrate, or TS, or intermediate. For the binding energy, one should calculate the energy of enzyme without the substrate in the active site. At the same time, the energy of free substrate should be calculated. The energy difference between enzyme bound with substrate and free enzyme and free substrate should give an approximate binding energy.

10. Page 9, line 4 from the bottom: “compared to the C5 of L-proline”

**Author response:**

As suggested, it was corrected.

11. The illustration in Figure 6, panel A seems to show that the hydride transfer from proline to the FAD N5 does not follow a linear path. What is the direct distance between the C5 of proline and the N5 of FAD? Is the purpose of this figure to show the projection of the hydride from the N5 of FAD or is it supposed to show the hydride being shared between the C5 and N5 atoms?

**Author response:**

The distance between the C5 of proline and the N5 of FAD is 2.55 Å. The hydride ion in A (TS2) is partially bonded to both C5 and N5. However, it is closer to N5 than C5. Yes, the purpose of figure is to show that the hydride being shared between the C5 and N5 atoms. The hydride transfer does not follow a linear path from C5 to N5. This can be better visualized by IRC calculations.

12. Page 11, Line 6: The authors comment “Based on this observation, it can be inferred that the release of iminoproline from the active site may occur after the oxidation of reduced FAD.” If this happened, it would imply a ternary mechanism, with P5C and ubiquinone bound to the enzyme simultaneously. This conclusion would contrast with experimental data showing a ping-pong mechanism. The authors perhaps should modify this statement or remove this conclusion until they conduct computational studies of the oxidative half-reaction.

**Author response:**

As suggested, this statement was removed.

13. In regards to the PC2 complex shown in Figure 6A, can the authors provide further insights into the possibility of the reverse step of hydride transfer from reduced FAD to iminoproline, thereby generating proline and oxidized FAD? What activation barrier would there be for this? Besides implying covalent bond formation with the N5, could the PC2 complex also imply the possibility of the reaction being reversible in the absence of ubiquinone?

**Author response:**

We have already calculated the activation barrier for the reverse process in Table 1 labeled as Ear(kcal/mol). In general, the reverse hydride transfer requires less barrier than the forward one based on our calculations. It is possible that in the absence of ubiquinone, the hydride transfer can be reversible. However, our calculations cannot predict any conformational change around iminoproline following the hydride transfer from L-proline to FAD. It gives only the snapshots of enzyme-substrate, enzyme-TS, and enzyme-product complex. It is quite possible that following the hydride transfer process from L-proline to FAD, some conformational change trigger the release of iminoproline from active site. In this respect, a more suitable way to study what happens after the hydride transfer process is MD simulations. The trajectory of MD simulation can provide better insights into what might happen to iminoproline following the hydride transfer process. Unfortunately, we don’t have expertise in MD simulations. But in future we plan to collaborate with a group having such expertise. We have modified the discussion based on your invaluable input.

14. Page 10, lines 10-12. Reference 38 is used to support the possibility of N5 nucleophilic attack on substrates. In addition to this review, a specific example of N5 attack on a covalent inhibitor for a ProDH enzyme is provided by the following study:Srivastava D, Zhu W, Johnson WH Jr, Whitman CP, Becker DF, Tanner JJ. The structure of the proline utilization a proline dehydrogenase domain inactivated by N-propargylglycine provides insight into conformational changes induced by substrate binding and flavin reduction. Biochemistry. 2010 Jan 26;49(3):560-9. doi: 10.1021/bi901717s. PMID: 19994913; PMCID: PMC3727237.This reference would be useful to include as well.

**Author response:**

As suggested, the reference was added.
